# A Redescription of the Bulla, Antennae, and Mouth Parts of Female *Clavella* sp. (Copepoda: Siphonostomatoida: Lernaeopodidae) Infesting Wild *Gadus morhua* Using Scanning Electron Microscopy (SEM)

**DOI:** 10.1155/2020/8891448

**Published:** 2020-12-22

**Authors:** Harry M. Murray, Jacqueline M. Hanlon, Kimberly Marshall, Corey Morris

**Affiliations:** ^1^Aquaculture, Biotechnology and Aquatic Animal Health Section, Fisheries and Oceans Canada, 80 East White Hills Road, PO Box 5667, St. John's, NL, Canada A1C 5X1; ^2^Ecological Science Section, Fisheries and Oceans Canada, 80 East White Hills Road, PO Box 5667, St. John's, NL, Canada A1C 5X1

## Abstract

Many members of the Copepod family Lernaeopodidae are well-known parasites of gadids. This study reports on the occurrence of a lernaeopodid infestation of wild-sourced *Gadus morhua* sampled from separate inshore (Gilbert Bay, NL) and offshore (Virgin Rocks, NL) populations from Newfoundland, Canada. The majority of the parasites were observed to be associated with the buccal cavity, gill filaments, gill arch, and occasionally near the outer edge of the operculum. Anatomical analysis and detailed redescriptions of the parasite's functional anatomy (mouth parts, antennae, and bulla complex) using high-resolution SEM indicated that the parasite was most likely of the genus *Clavella*. New morphological details of the second antennae ornamentation, first maxillae, bulla complex, and the oral cone are provided and discussed with regard to their potential in taxonomic applications.

## 1. Introduction

Copepods are small, abundant, inconspicuous aquatic crustaceans. Approximately 11 500 species are known, half of which are symbiotic with most of those being parasitic and exhibiting a broad host specificity [1]. Members of the copepod family Lernaeopodidae are parasitic mainly on marine fish, including both Selachii and Teleostei [2]. Boxshall [3] reported up to 45 different genera from this family parasitic on teleosts and some elasmobranch fishes. As is common in parasitic copepods, the Lernaeopodidae display an unusual sexual differentiation. Only the female is attached as a parasite on the host. The small male lives in temporary association, often on the body of its partner. The female generally utilizes a characteristic chitinous attachment organ called the bulla to attach to the host permanently [2, 4].

The copepod parasites (including the lernaeopodids) of the gadids are generally well known, partly as a result of the large amount of material collected from commercial fisheries [5, 6]. One of the best illustrated examples is the specific case of Atlantic cod, *Gadus morhua*. Hemmingsen and MacKenzie [7] list 107 named parasite species from ten separate phyla; of these, seven were suggested to be specific to Atlantic cod, 17 to gadoids generally.

The genus *Clavella* contains approximately 19+ species [4]. Some of which are specific to gadids [8] with early taxonomic descriptions dating back to the early 20^th^ century [9–13]. The majority of the anatomical descriptions of *Clavella* sp. are based on detailed drawings produced through the use of basic light microscope techniques. Few advanced scanning electron microscopy (SEM) anatomical investigations have been reported for this group. Benkirane et al. [2] surveyed the structural variability of the bulla complex in a range of different species of lernaeopodids including *Clavella adunca*.

In the present study, we describe a lernaeopodid parasite isolated from wild-sourced Atlantic cod, sampled from both inshore (Gilbert Bay, NL) and offshore (Virgin Rocks, NL) North Atlantic populations. In order to identify and better describe the parasite, we used high-resolution scanning electron microscopy (SEM) techniques to resolve previously undescribed detail of the antennae, mouth parts, and the bulla complex to potentially help in assisting new taxonomic applications.

## 2. Materials and Methods

Forty-five *Gadus morhua* were captured using hook and line from two locations in Newfoundland and Labrador, inshore at Gilbert Bay and offshore at the Virgin Rocks, between June and October 2018. Fish were subsequently transported in tanks equipped with supplemental oxygen and introduced to a flow through ambient tank facility located at the Northwest Atlantic Fisheries Centre, Fisheries and Oceans Canada, located in St. John's NL. In December 2019 during routine measurements, a number of fish independent of origin were noted to present with a presumptive parasitic copepod infestation.

In order to determine basic taxonomy and gross morphological detail of the observed parasites, a small sample of approximately 50 individuals were randomly removed from infested fish with fine forceps, placed in glass vials containing 10% neutral buffered formalin (NBF), and stored at 4°C until further analysis. Fixed parasites were photographed using a Leica S9i dissecting microscope with attached camera (Leica Microsystems, Wetzlar, Germany). Individual specimens were temporarily mounted on a plastic composite black plate for photography. All individuals were deemed to be of the same or similar species. Anatomical measurements were reported as the mean ± SD of 50 individuals. An additional sample of fixed specimens was dehydrated for scanning electron microscopy (SEM) according to the protocol outlined in [14]. In brief, individuals were washed 3 times in 1x Dulbecco's phosphate buffer solution followed by 3 further washes in distilled water, rinsed in 0.1% Tween 80, and washed 3x more in distilled water. Specimens were then rapidly passed through an ethanol series (50, 70, 95, 2x 100). After the last 100% wash, ethanol was replaced with 2 ml of hexamethyldisilane (HMDS) swirled briefly and allowed to sit for 2 minutes. The HMDS was removed and replaced by 1.5 ml of fresh reagent and stored in an evacuated desiccator overnight.

Dehydrated individual parasites were subsequently arranged on flat SEM stubs fitted with two-sided tape and ion sputter coated with gold (Anatech Hummer 6.2) in preparation for observation with a Hitachi SU-1510 scanning electron microscope set at 15 kV.

## 3. Results

**Figure 1:**
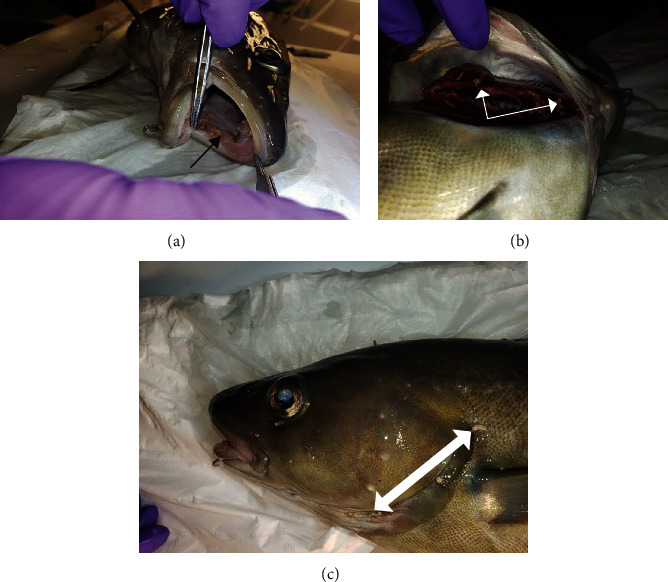
Anatomical distribution of presumptive *Clavella* sp. infesting *Gadus morhua.* (a) Oral cavity (black arrow). (b) Gill filaments and arch (white arrows). (c) External operculum (black arrows).

Of the 50 individual parasites sampled across the two fish populations for observation, all were noted to be ovigorous female lernaeopodid copepods. The majority of specimens were distributed through the oral cavity and gills (Figure 1).

**Figure 2:**
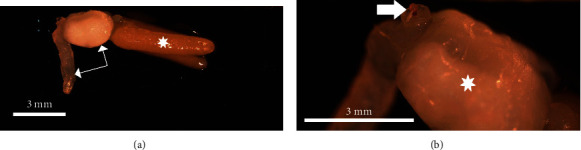
Overall anatomy of female presumptive *Clavella* sp. sampled from *Gadus morhua*. (a) Anatomical demonstration of cephalothorax (horizontal white arrow) and trunk (vertical white arrow). Egg strings (asterisk). (b) The bulla complex (white arrow) and trunk (asterisk).

The average parasite body length from the tip of the cephalothorax to the genital process was 9.0 mm ± 0.05. Egg strings were 5.02 ± 0.1 mm giving the total average length for a gravid female as 14.02 ± 0.11 mm (Figure 2). The mean long axis of the trunk was 2.0 ± 0.06 mm. The cephalothorax was oriented perpendicular to the long axis of the trunk, extending ventrally and laterally. It was cylindrical and approximately 1.75x the length of the trunk (3.49 ± 0.06 mm) with an average width of 1.03 ± 0.07 mm through most of its length tapering to 0.50 ± 0.01 mm near the terminus (cephalic shield). The dorsal end of the cephalothorax (second maxillae) terminated with a bulla, manubrium, and associated collar (Figure 2). The genital process was elongated not lobed and averaging about 0.49 ± 00 mm in length.

### 3.1. The Bulla, Manubrium, and Collar

**Figure 3:**
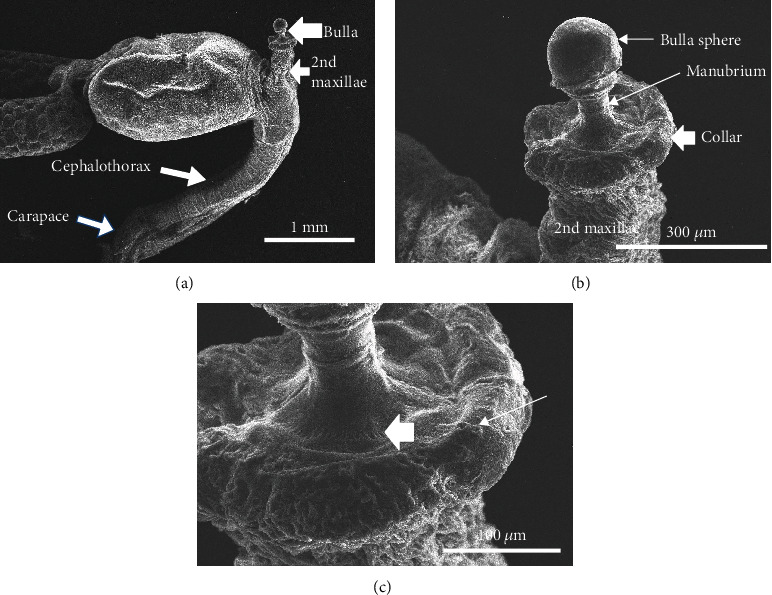
SEM bulla complex. (a) Overall view of trunk and cephalothorax. White large arrowhead indicates position of bulla complex with sphere, manubrium, and collar. Modified second maxilla supports the bulla complex. Note elongated cephalothorax and associated carapace. (b) Note detail of bulla complex showing the bulla sphere, manubrium, and collar supported by the second maxilla. (c) High-resolution detail of the manubrium and collar showing detail of manubrium ornamentation at base (large arrowhead) and pores on the surface of the collar (thin arrow).

The dorsal terminus of the cephalothorax (second maxillae) was characterized by the bulla attachment organ. The structure was observed to be composed of three parts: the bulla sphere, stem or manubrium, and the collar (Figure 3(a)). The bulla sphere was observed to be a rounded relatively smooth structure with no obvious ornamentation sitting on a robust manubrium extending approximately 150 *μ*m from the base of the sphere to the collar (Figure 3(b)). The base of the manubrium at its connection with the collar is decorated with flat ornate comb-like ridges (Figure 3(c)). The collar is a thick rounded structure supporting the manubrium and the associated sphere (Figure 3(c)). The surface of the collar contained numerous small raised pores some of which appear as if they are associated with a thickened layer (Figure 3(c)).

### 3.2. The Antennae and Mouthparts

**Figure 4:**
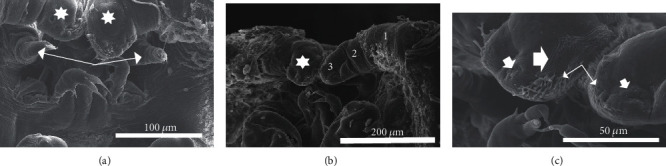
(a) Overall view of antenna positioning. Note primary antennae (arrows) and secondary antennae (asterisks). (b) Basic structure of secondary antennae showing 3 segment pattern. Asterisk indicates terminal segment of antennae. (c) Detail of the terminal segment of second antenna. Note microridges (large arrowhead), flexible spines (thin arrows), and sensory pits (small arrowhead).

The ventral terminus of the cephalothorax contains the primary and secondary antennae as well as components of the buccal apparatus. The primary antennae are paired and positioned to the left and the right but slightly below the oral cone, narrowing from a stout base and gradually tapering to a tip characterized by five blunt and flexible spines extending from terminal tubercles (Figure 4(a)). The secondary antennae are paired large blunt uniramous structures with three segments and positioned immediately above and adjacent to the oral cone notably obscuring it at times (Figures 4(a)–4(c)).  Figure 4(c) illustrates the ornamentation of the secondary antennae. The blunt tip is characterized by numerous microridge-like structures oriented laterally toward the oral cone and forming a complex pattern. Additional ornamentation includes numerous flexible spine-like projections which frequently appear flattened to the surface of the blunt tip. A cluster of pit-like structures were also noted on each antenna and were oriented toward the oral cone (Figure 4(b)).

**Figure 5:**
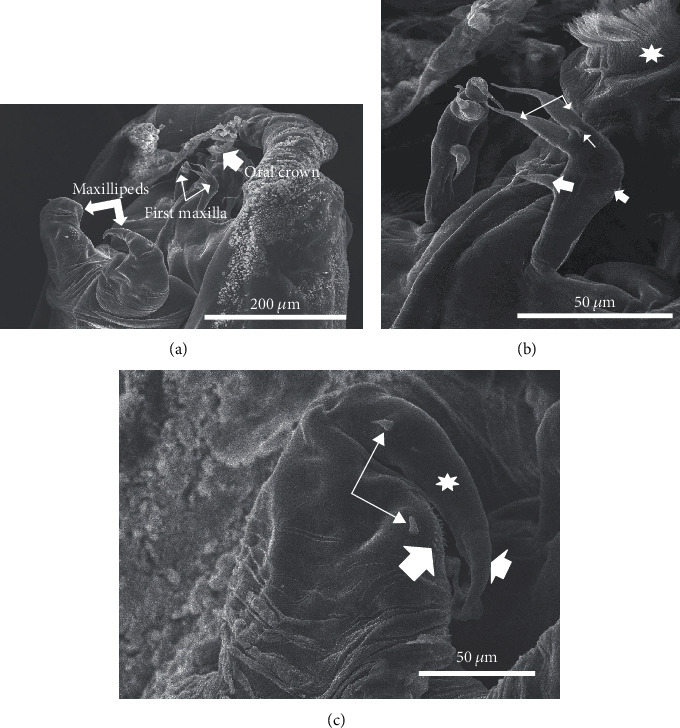
(a) Overall view of the anterior terminus of the cephalothorax including the mouth parts (2^nd^ maxillipeds (large arrows), 1^st^ maxilla (small arrows), and oral crown with apical setae (large arrowhead)). (b) Detail of the 1^st^ maxillae. Note endopod and exopod with long slender spines and situated at the apex (large arrows). Large arrowhead indicates a short palp with primary and secondary spines. Note short stout spine at the base of the exopod (small arrow) near small patches of spicules (small arrowhead). Star indicates oral setae. (c) Maxilliped with larger jointed subchela (star) with claw and pincers (small arrowhead). Note short robust spines (arrows) and small ornamental denticles (large arrowhead).

The buccal apparatus consists of paired first maxillae and maxillipeds situated ventral to the oral cone (Figure 5(a)). The first maxillae are club-shaped with a rounded apex, decorated with patches of small spicules along the outer curve of the club (Figure 5(b)). The apex possesses an endopod and an exopod, each with long and slender flexible spines. At the base of the exopod and on its lateral side, there is a short stout spine. Additionally, a short palp is located about halfway down the structure on the ventral side and contains a primary and secondary spine (Figure 5(b)).

The maxillipeds are large, prominent, and paired structures situated immediately below the first maxillae and are characterized by a large jointed subchela terminating in a robust claw possessing two pincers (a larger upper and a smaller lower) (Figures 5(a) and 5(c)). Two short robust spines are present, one located near the inner curve adjacent to two rows of small ornamental denticles and a second on the subchela near the joint (Figure 5(c)).

### 3.3. The Oral Cone

**Figure 6:**
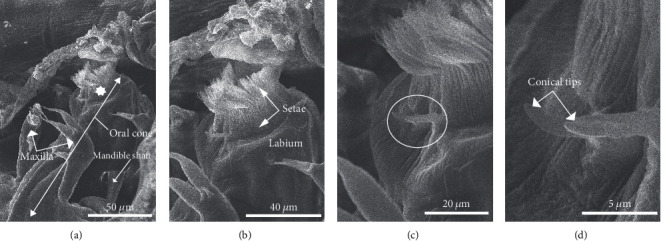
Detail of the feeding apparatus. (a) Oral cone (large double-headed arrow) with maxilla (arrows) and seta crowning the oral tube (star). Note the mandible shaft (arrow). (b) Detail of the oral crown and border of blade-like setae (arrows). Note adjacent supporting labium. (c) High-resolution image showing the oral crown with rigid conical setae (circle). (d) Further high-resolution detail of rigid setae demonstrating the conical nature of the tips (arrows).

The oral cone is situated centrally, along the ventral wall at the terminus of the cephalothorax (Figure 6(a)). Its anterior end terminates in a dense crown of setae supported by the labium below. Each individual seta has a narrow bottom half and a top half that is flattened and blade-like (Figure 6(b)). Additionally, two modified and shortened setae extend ventrally away from the crown and appear rigid (Figures 6(c) and 6(d)). The tips are blunt, conical, and raised (Figures 6(c) and 6(d)). In the absence of dissection, the mandibles were not observed in the individual preparations from this investigation although a possible mandibular shaft was noted.

## 4. Discussion

The occurrence of lernaeopodid parasites (Copepoda: Siphonostomatoida: Lernaeopodidae) in association with wild gadids is not unusual, with descriptive accounts extending back to the early 20^th^ century [12, 13, 15, 16] and with later descriptions and redescriptions provided by [4, 8, 17–19]. Some more recent surveys of this group have focused on the potential impact of such parasites on cultured or tank maintained gadids. Bricknell et al. [5] and later Heuch et al. [20] reported the relatively common occurrence of the lernaeopodid *Clavella* on farmed cod. Kahn [21] indicated that the pellelid copepod *Lernaeocera branchialis* can cause significant mortality in juvenile *Gadus morhua* especially following multiple infections. Additionally, environmental extremes including warm summer water temperatures were noted to induce mortality in Atlantic cod infested with the same parasite.

Morphological characteristics have historically been useful as metrics for classifying the taxonomy of parasitic copepods [8, 18, 22]. Within the present study, individual specimens exhibited many morphological features that aligned well with the lernaeopodid genus *Clavella* and more specifically *Clavella adunca* originally described from *Gadus callarias* by [8] and rereviewed by [17]. It is noteworthy however that in the present study the dimensions of the trunk and cephalothorax were slightly larger here than those reported in [20] but comparable to those of a more recent study by Gjøsæter [23] who surveyed *Clavella adunca* from Polar cod, *Boreogadus saida*. Kabata [8] noted that many appendages (i.e., antennae and mouth parts) are mobile and their appearance can vary depending on how they were prepared. This may explain the slight morphological variations observed here where specimens were chemically fixed in formalin as opposed to traditional preservation in ethanol and clearing in lactic acid [24].

Many anatomical descriptions of lernaeopodids including *Clavella* were traditionally based on individually fixed dissections, cleared in lactic acid and illustrated from camera lucida-assisted drawings with phase-contrast illumination and interference techniques for finer features [8, 18, 22, 25]. In the present study, the application of high-resolution SEM allowed for new visualization of distinct anatomical features of the secondary antenna, the bulla complex, and components of the oral cone.

SEM is well accepted as an effective tool for study of the morphological characteristics of copepods and small related crustaceans, including the parasites [2, 14, 25–30]. Traditional SEM preparation protocols are known to be particularly rigorous and time-consuming and thus not always considered routine for the quick preparation of small delicate organisms due to their structural complexity and relatively delicate anatomy [14, 26]. Proper fixation and careful but rapid dehydration are key to preserving fine structure. Dippenaar and Jordaan [30] and later Murray et al. [14] used hexamethyldisilazane (HMDS) for the rapid dehydration of small parasitic copepods specifically for SEM examination. Both studies showed excellent preservation of the fine detail of the mouth and antenna structures of Naobranchids and Ergasilids, respectively.

Interestingly, of those studies utilizing SEM to investigate parasitic copepods, few have focused on members of the Lernaeopodidae. Some of those that did focus on this group include Van Niekerk and Olivier [29], who describe a new species of lernaeopodid, *Alella gibbosa*; Robinson and Avenant-Oldewage [31], who investigated aspects of the morphology of *Lernaea cyprinacea*; Benkirane et al. [2], who studied the variability in the structure of the bulla attachment organ from a range of lernaeopodid species including *Clavella adunca*; and later Dippenaar and Jordaan [30], who investigated the overall anatomy of the male and female *Naobranchia kabatana n.* sp. Most recently, Ruiz et al. [32] utilized SEM in combination with light microscopy and molecular analysis to characterize members of the lernaeopodid genus *Salmincola* from brook trout. The SEM preparation techniques utilized in the present study including the rapid dehydration technique outlined in [14] provided high-resolution descriptions of the antennae, mouth parts, and the bulla attachment organ of a presumptive *Clavella* species sampled from *Gadus morhua*.

The primary antennae associated with the specimens in the present study were narrowed, extending from a stout base and tapering to a tip characterized by four or five blunt and flexible spines. While there was some difficulty in getting a clear image of each spine, the primary antennae here are reminiscent of the primary antennae from *Clavella stellate*, a parasite of hake [33], or *Clavella bowmani*, originally described as a parasite of *Notothenia* [18].

Kabata [18] also provided a detailed multispecies comparison of the secondary antennae of the *Clavellinae* from a taxonomic perspective, exploring the structural variability across seven species. In the present study, the secondary antennae were generally reminiscent of the *Clavellinae* and noted to be robust uniramous cylindrical structures composed of 3 segments including a blunt jointed terminal segment with associated armament. Distinct segmentation varies across species based on the descriptions provided in [18] with only 5 of the 8 species described as having 3 distinct segments.

The armament of the terminal segment of the secondary antennae described here consisted of numerous flexible spines that in the SEM preparation consistently appeared flattened to the surface of the segment and could be speculated to have a sensory function. Additional ornamentation included two short denticles oriented toward the oral crown. Rows of microridges and the sensory pits decorating the ventral side of the terminal segment have to the best of our knowledge not previously been described from this group and could provide a new taxonomic tool.

The maxillae and maxillipeds are prominent feeding structures of the oral complex in parasitic copepods. Kabata [8] provided a description of adult male and female specimens from an unknown species of *Clavella* sampled from the gills of English whiting (*Merlangius merlangus*) including direct comparisons to *Clavella adunca f. sciatherica* and *Clavella dubia* previously provided by [17]. The descriptions provided in the present study for the 1^st^ maxillae and maxillipeds were very comparable to those included for the above species. The only variation from the Kabata [8] description of the first maxillae was the presence of two patches of very fine spicules along the outer curve of the club. These may very likely only be visible using the higher resolution capabilities of the SEM. Similarly, the maxillipeds described from the Kabata [8] specimens were close structurally to those observed in the present study. In this case, the only variation was the observed absence of the serrations on the lower margin of the claw in the SEM preparations. While it was generally possible to observe the structures from multiple angles on different specimen preparations, the serrations were never observed in the present study.

Within the lernaeopodid group, the 2^nd^ maxillae undergo a developmental modification toward the posterior end of the cephalothorax forming the attachment organ or bulla complex which is used to procure a sure anchorage into the host tissue. Surprisingly, few detailed studies of the lernaeopodid bulla complex are available. One of the earliest descriptions from this group was provided for *Clavella iadda* and *Clavella sciatherica* by Leigh-Sharpe [16]. He included drawings of the bulla from both species and described it as being ovate, spherical, or cylindrical but did not provide descriptions of the collar or manubrium. Kabata and Cousens [34] discussed the bulla complex generally and reported that variation in structural configuration was related more to the type of host tissue to which it was attached then the phylogeny of the parasite. Additionally, they observed that the bulla was more than an attachment organ as it appears to enter into a physiological association with the tissue of the host. Much more recently, Benkirane et al. [2] applied SEM techniques to provide a comprehensive review of the structure of the bulla attachment organ from 18 different species of Lernaeopodidae and showed considerable structural variability within the group generally but indicated good morphological stability within a genus or species. It was unclear as to whether the morphological variability was due to taxonomical differences or modifications specific to the tissue in which it was imbedded. In the present study, the majority of parasites were noted to be attached to the buccal cavity, gills, arch, and occasionally near the outer edge of the operculum. The descriptions provided by Benkirane et al. [2] for *Clavella adunca* and related species specifically indicated a clear spherical bulb structure for the bulla with additional surface ornamentation. These observations were similar to that for the bulla sphere in the present study although the ornamentation details described in Benkirane et al. [2] were not as evident here. Additionally, Benkirane et al. [2] found that the length of the manubrium was variable across species and may also be linked to preferred host tissue structure. In the present study, the manubrium was distinct and showed comb-like ornamentation at the transition with the collar. The collar itself was thick with numerous pores through its surface. This characteristic was not mentioned in previous studies. Kabata [33] did describe the presence of as many as 18 ducts extending from the manubrium and collar into the bulla of *Clavella stellata*. It is possible that these ducts if present may be related to the pore structures observed in the bulla collar from the present investigation. Physiologically, it is not clear from either study as to their function and further work is necessary.

Few if any detailed descriptive studies of the oral cone have been completed for species within the subfamily *Clavellinae*. However, early observations of the oral cone of lernaeopodids generally extend back to the Kabata [33] study of the mouth and mouthparts of *Lernaeocera branchialis*. Subsequent studies investigated the functional morphology of the mouth tube from a number of related species using basic microanatomical techniques [35–38]. More recently, Chandran and Nair [37] and Chandran [39] both provided detailed histological studies of the structure and musculature of the mouth tube (oral cone) from two species of lernaeopodid (*Pseudocharopinus narcinae* and *Isobranchia appendiculata*, respectively). Van Niekerk and Olivier [29] used SEM techniques to describe the mouth tube and related structures of the lernaeopodid *Alella gibbosa* including the orientation of the labium and labrum. More recently, Ruiz et al. [32] used SEM to provide a new description of the anatomy of an lernaeopodid from the genus *Salminocola* infecting trout including that of the oral cone.

To the best of our knowledge, the present study is a first to provide a high-resolution description of the oral cone from an individual within the subfamily *Clavellinae*. The crown was characterized by a dense ring of blade-like setae sitting between the labia and as such was very similar to that described by Chandran [39] for the lernaeopodid *I. appendiculata* and for the *Salminocola* sp. described in Ruiz et al. [32]. The two ridged setae extending out from the base of the crown and the ventral lip of the labium appear to have not been described previously. Their specific function is unknown. The mandible was not observed in these preparations as they are typically located in the oral tube itself and would not be visible in a whole-mounted preparation as is the case here. The mandible shaft did appear to be visible at its insertion point on the dorsal and lateral side of the oral tube however and thus similar to that noted in [29] for *A. gibbosa* where the tip of the mandible was occasionally visible within the oral cavity. Ruiz et al. [32] also noted the tip of the mandible in the oral cavity of the mouth tube. Unlike that observed in Van Niekerk and Olivier [29] and Ruiz et al. [32], the rostrum and labrum were not visible in the current preparations. It is unclear as to whether these are in fact distinct structures in the *Clavellinae*.

In summary, the present study records the observation of a lernaeopodid parasite infestation among a sample of wild-sourced *Gadus morhua* and provides a redescription of the anatomy with a focus on the oral appendages, bulla, and manubrium. Final presumptive identification of the parasite was supported by high-resolution SEM of the functional anatomy and supported the conclusion that the parasite was of the lernaeopodid genus *Clavella*.

## Data Availability

The full extent of the microscopy and numerical data used to support this study are included within the article.
